# An EGF- and Curcumin-Co-Encapsulated Nanostructured Lipid Carrier Accelerates Chronic-Wound Healing in Diabetic Rats

**DOI:** 10.3390/molecules25204610

**Published:** 2020-10-10

**Authors:** Hye-Jin Lee, Moses Jeong, Young-Guk Na, Sung-Jin Kim, Hong-Ki Lee, Cheong-Weon Cho

**Affiliations:** 1 College of Pharmacy and Institute of Drug Research and Development, Chungnam National University, 99 Daehak-ro, Yuseong-gu, Daejeon 34134, Korea; haejin101@naver.com (H.-J.L.); moses1korea@gmail.com (M.J.); youngguk@cnu.ac.kr (Y.-G.N.); lanop@naver.com (S.-J.K.); 2 Animal Model Research Group, Jeonbuk Branch, Korea Institute of Toxicology (KIT), Jeongeup, Jeollabuk-do 53212, Korea

**Keywords:** EGF, curcumin, nanostructured lipid carrier, chronic-wound healing, antioxidant effect

## Abstract

Nanostructured lipid carriers (NLC) are capable of encapsulating hydrophilic and lipophilic drugs. The present study developed an NLC containing epidermal growth factor (EGF) and curcumin (EGF–Cur-NLC). EGF–Cur-NLC was prepared by a modified water-in-oil-in-water (w/o/w) double-emulsion method. The EGF–Cur-NLC particles showed an average diameter of 331.8 nm and a high encapsulation efficiency (81.1% and 99.4% for EGF and curcumin, respectively). In vitro cell studies were performed using two cell types, NIH 3T3 fibroblasts and HaCaT keratinocytes. The results showed no loss of bioactivity of EGF in the NLC formulation. In addition, EGF–Cur-NLC improved in vitro cell migration, which mimics the wound healing process. Finally, EGF–Cur-NLC was evaluated in a chronic wound model in diabetic rats. We found that EGF–Cur-NLC accelerated wound closure and increased the activity of antioxidant enzymes. Overall, these results reveal the potential of the NLC formulation containing EGF and curcumin to promote healing of chronic wounds.

## 1. Introduction

Wound healing is an essential physiological process and generally consists of four phases: hemostasis, inflammation, proliferation, and tissue remodeling [1]. After wounding, hemostasis begins with the formation of a fibrin clot. Next, pro-inflammatory cytokines and growth factors, in particular, epidermal growth factor (EGF), are released around the wounded tissues [2]. In the inflammatory phase, inflammatory cells, such as neutrophils, macrophages, and lymphocytes reach the wound [3,4,5]. At the same time, the proliferative phase starts, during which epithelial proliferation and migration occur. In particular, fibroblasts produce collagen for extracellular matrix (ECM) formation [6]. In the final step, the remodeling phase, the granulation tissue is remodeled, reestablishing the normal tissue architecture [6].

EGF is the key regulator of cell proliferation and differentiation and stimulates the migration and proliferation of fibroblasts and keratinocyte, as well as collagen deposition. It is known that EGF binds to the EGF receptor, promoting DNA synthesis and cell proliferation [7].

Therefore, providing EGF to wounds leads to a rapid re-epithelization and reduces the risk of infection [8,9,10]. Many attempts have been made to use EGF for the treatment of wounds, burns, and diabetic ulcers [9,11]. However, wound sites contain hydrolytic enzymes, which degrade extracellular matrix proteins [12]. Therefore, a strategy to prevent degradation is needed to successfully apply EGF to chronic wound.

A diabetes-induced chronic wound is characterized by chronic inflammation. Chronic inflammation caused by oxidative stress leads to cell apoptosis [13,14,15]. Oxidative stress is defined as an unbalance between excess production of reactive oxygen species and antioxidant defenses [16]. Curcumin is a lipophilic compound and bifunctional antioxidant that can react directly with reactive oxygen species and induce the production of antioxidant enzymes [16]. Therefore, it has been reported as a wound healing agent [17,18]. Moreover, it was shown that curcumin has anti-inflammatory and anti-bacterial effects [17,18,19]. However, the clinical application of curcumin has been hampered by its low water solubility and stability [19], which cause a low bioavailability. Nanostructured lipid carriers (NLC) are suitable carriers to overcome these limitations.

Solid lipid nanoparticles (SLN) were developed in 1991 as a nanocarrier system [20]. After that, NLC were introduced as an alternative to overcome the limitations of SLN (e.g., low loading capacity). NLC are known as efficient and non-toxic drug delivery systems for a variety of active compounds [21,22]. It has been reported that NLC protect drugs from oxidation or hydrolysis and provide their controlled release [23]. NLC can be administered via various routes (e.g., oral, parenteral, etc.). In particular, NLC are suitable for topical administration because they can provide an appropriate drug concentration in the skin [24,25]. All these characteristics make the use of lipid nanoparticles a suitable strategy for chronic-wound healing.

In this study, an EGF- and curcumin-co-encapsulated NLC (EGF–Cur-NLC) was prepared by a water-in-oil-in-water (w/o/w) double-emulsion method. In vitro cell studies were performed using NIH 3T3 fibroblasts and HaCaT keratinocytes. A proliferation assay was carried out to evaluate the suitability of the NLC as a carrier for EGF. A migration assay confirmed the potential of EGF–Cur-NLC as a wound-healing agent. Then, EGF–Cur-NLC was applied topically to streptozotocin-induced diabetic rats, a chronic-wound model. The wound healing properties of EGF–Cur-NLC were confirmed by measuring the wound closure rate and the level of antioxidant enzymes.

## 2. Results and Discussion

### 2.1. Optimization of EGF–Cur-NLC

**Figure 1:**
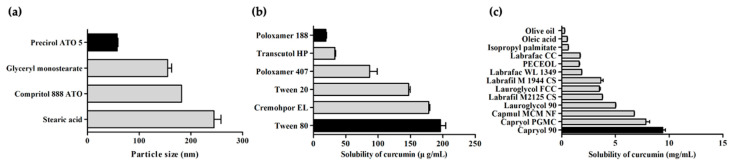
Particle size of solid lipid nanoparticles prepared with various solid lipids (**a**); solubility of curcumin in various liquid lipids (**b**) and 1% surfactant solutions (**c**). The data are shown as mean ± SD (*n* = 3).

The type of lipids and surfactants, which is a major component of NLC, determines the physicochemical properties of NLC. Thus, lipids and surfactants were screened to optimize EGF–Cur-NLC. The selection of a solid lipid was based on particle size (Figure 1a). A small-particle dispersion is kinetically stable during sedimentation [26]. Because of this, solid lipids were evaluated on the basis of their particle size when preparing nanoparticles. We found that the solid lipid Precirol^®^ ATO 5 (57.3 ± 1.4 nm) presented the smallest particles.

Curcumin has poor aqueous solubility. Therefore, it is important to select an appropriate liquid lipid to ensure its solubilization. Among liquid lipids, curcumin showed high solubility in the semisynthetic modified oil Capryol^®^ 90 (9.40 ± 0.36 mg/mL), as shown in Figure 1b. In contrast, it showed low solubility in long.chain unmodified oils (oleic acid and olive oil, 0.50 ± 0.06 mg/mL, 0.27 ± 0.03 mg/mL, respectively). Thus, Precirol^®^ ATO 5 and Capryol^®^ 90 were selected as solid and liquid lipids for NLC preparation.

The surfactant was selected by a solubility test which is conducted by dissolving the materials in a 1% surfactant solution (Figure 1c). Surfactants exhibiting low drug solubility affect the binding of the drug to a lipid matrix. As a result, the use of a surfactant with low drug solubility increases the amount of drug encapsulated in the lipid core of a lipid carrier [27]. Poloxamer 188 (19.17 ± 2.35 μg/mL) and Tween 80 (196.29 ± 14.08 μg/mL) showed the lowest and highest solubility of curcumin, respectively. It was reported that Tween 80 enhances the stability and uniform distribution of particles [28]. Also, Patel et al. suggested that the combination of two surfactants provides good stability and small particle size [29]. Therefore, Poloxamer 188 and Tween 80 were chosen as surfactant and co-surfactant for NLC preparation.

The physicochemical properties of different EGF–Cur-NLC formulation were examined to establish the optimal formulation. The EGF–Cur-NLC was prepared by a modified w/o/w double-emulsion method. EGF–Cur-NLC with different ratios of solid and liquid lipids were prepared. The amounts of Precirol^®^ ATO 5 and Capryol^®^ 90 ranged from 50 to 70 mg and from 30 to 50 mg, respectively. The molten lipid mixture with the EGF solution was placed in a 40 °C water bath to form a water/oil (w/o) emulsion. In these conditions, the lipid mixtures with ratios of 90:10 and 80:20 (Precirol^®^ ATO 5/Capryol^®^ 90) hardened, so this composition has been excepted for NLC preparation. The results showed that the particle size significantly increased when increasing the amount of Capryol^®^ 90. This result is similar to those reported by Sangsen et al. [30]. This indicates that the particle size increases due to a more disordered crystalline structure inside the nanoparticles [30]. Therefore, further studies were performed using formulation 1 (F1), which is consists of Precirol^®^ ATO 5 and Capryol^®^ 90 in the ratio of 70:30.

The optimal conditions for EGF–Cur-NLC preparation were determined on the basis of the physicochemical properties of the resulting EGF- and curcumin-loaded NLC formulations (Table 1). NLC formulations with different ratios of Precirol^®^ ATO 5 and Capryol^®^ 90 were prepared, fixing the amount of the aqueous phase and the ratio of surfactants at 5 mL and 1%, respectively (*w*/*v*, poloxamer 188 0.5% and Tween 80 0.5%). We found that F1 (331.8 ± 29.1 nm) had smaller particle size than the other formulations (394.3 ± 17.5 nm for F2 and 509.2 ± 18.3 nm for F3). The polydispersity index (PDI) was similar for all formulations (F1–F3). In addition, the drug encapsulation efficiency (EE) was similar for all formulations. The zeta potential of F1 was −6.64 ± 0.51 mV. The size of EGF–Cur-NLC was around 300 nm; according to the literature, the usual size of NLC ranges from 10 to 1000 nm [31]. Therefore, the F1 formulation was selected for further studies based on the particle size.

### 2.2. Crystallinity

**Figure 2:**
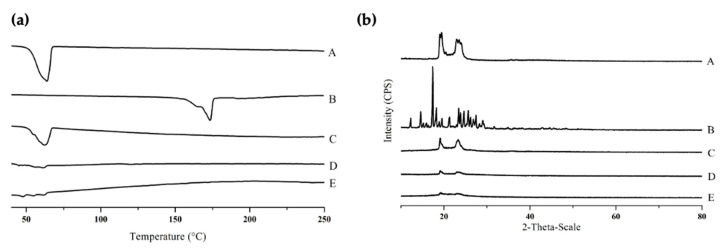
DSC thermograms (**a**) and XRD patterns (**b**) of (A) Precirol^®^ ATO 5, (B) free curcumin (Cur), (C) physical mixture, (D) blank nanostructured lipid carrier (NLC), (E) epidermal growth factor (EGF)–Cur-NLC.

Figure 2a shows the differential scanning calorimetry (DSC) thermograms of Precirol^®^ ATO 5, free curcumin, the physical mixture, blank NLC, and EGF–Cur-NLC. The thermogram of Precirol^®^ ATO 5 and free curcumin showed peaks at 56 °C and 183 °C, respectively. These peaks were disappeared in the thermogram of EGF–Cur-NLC, so it indicates the curcumin dissolution and NLC formation. In addition, EGF–Cur-NLC showed an increased number of endothermic peaks under 56 °C compared to blank NLC.

In XRD analysis, the intensity of Precirol^®^ ATO 5 peaks was reduced in the NLC formulation (Figure 2b). In addition, the intensity of the peaks corresponding to EGF–Cur-NLC was lower than that of the peaks for blank NLC. In DSC analysis, it was observed the absence of endothermic peaks for EGF–Cur-NLC. This observation indicates that the drug was not in a crystalline state but had an amorphous or molecularly dispersed structure in the lipid matrix [32]. The number of defects in the lipid crystal lattice increased with the incorporation of drug and oil inside the solid lipid matrix [33]. This result could be attribute to the method (w/o/w double emulsion) for preparing the NLC formulation. In XRD analysis, the intensity of the EGF–Cur-NLC peaks decreased when decreasing the crystallinity of the lipid matrix. This result indicate that the incorporation of the drug in the lipid matrix increased the imperfections of the crystals. Therefore, these results demonstrate that curcumin was molecularly dispersed in the lipid matrix, which improved its solubility.

### 2.3. In Vitro Release Study

**Figure 3:**
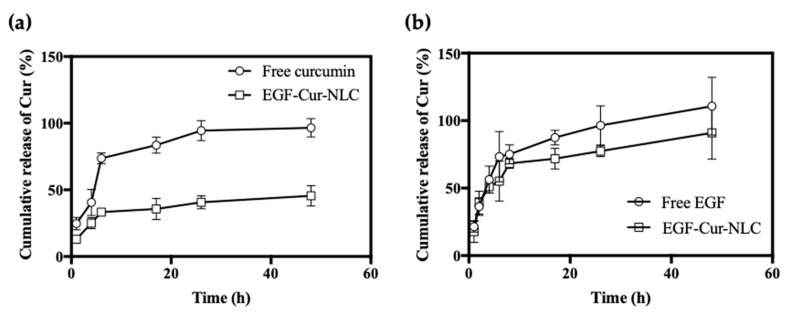
In vitro drug release profile of (**a**) curcumin form free curcumin and EGF–Cur-NLC in 50% ethanol solution, (**b**) EGF from free EGF and EGF–Cur-NLC in PBS solution at pH 7.4. The data are shown as mean ± SD (*n* = 3).

The pH of intact skin ranges from 4 to 6. However, when the skin is injured, it is exposed to the internal environment with pH 7.4. Therefore, we evaluated drug release in wounded skin at pH 7. for the free-drug formulation, the cumulative release rate of EGF and curcumin was approximately 90% within 24 h (Figure 3). In contrast, for the EGF–Cur-NLC formulation, the release of EGF showed a biphasic release profile. Curcumin presented a burst release corresponding to approximately 25%, followed by a sustained released in 48 h. Controlled-release formulations are beneficial, especially for the treatment of chronic wounds, as they reduce the frequency of drug administration, thus increasing patient compliance [34]. In an in vitro release study, EGF in the EGF–Cur-NLC formulation showed a biphasic release pattern, with an initial burst release followed by a sustained release. It is suggested that the drug present on the particles’ surface underwent a burst release, while the encapsulated drug was released over a prolonged time [20]. The burst release of EGF is promote a rapid activation of the keratinocytes at the wound edge [35]. The drug release mechanism was evaluated by the Korsmeyer–Peppas model. The n values for EGF, Cur, EGF in EGF–Cur-NLC and Cur in EGF–Cur-NLC were 0.40, 0.37, 0.26, and 0.31, respectively (Table 2). This indicates that EGF and Cur in EGF–Cur-NLC showed a quasi-Fickian drug release mechanism.

### 2.4. Cell Proliferation and Migration Assays

**Figure 4:**
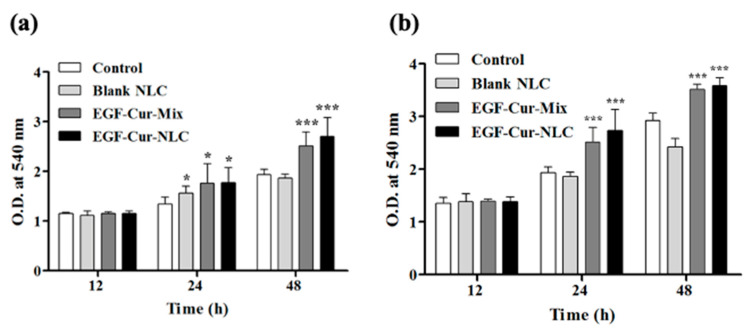
Cell proliferation assay for (**a**) NIH 3T3 fibroblast and (**b**) HaCaT keratinocytes using the MTT assay. The data are shown as mean O.D. ± SD (*n* = 6); * *p* < 0.05 and *** *p* < 0.001 were considered significant compared to the control.

To confirm the potential mitogenic activity of EGF in EGF–Cur-NLC, a cell proliferation assay was performed (Figure 4). Cells were treated with blank NLC at the same lipid concentration as EGF–Cur-NLC. The results showed that blank NLC was non-toxic in NIH 3T3 fibroblasts and HaCaT keratinocytes. Cells treated with a mixture of EGF and Cur (EGF–Cur-Mix) or with EGF–Cur-NLC exhibited increased cell proliferation than the control groups (treated with serum-free DMEM and blank NLC). To assess the level of activity of EGF when encapsulated in NLC, the mixture of EGF and curcumin and EGF–Cur-NLC, containing the same concentration of EGF, were compared. EGF–Cur-Mix and EGF_Cur-NLC showed no difference in proliferation rate.

**Figure 5:**
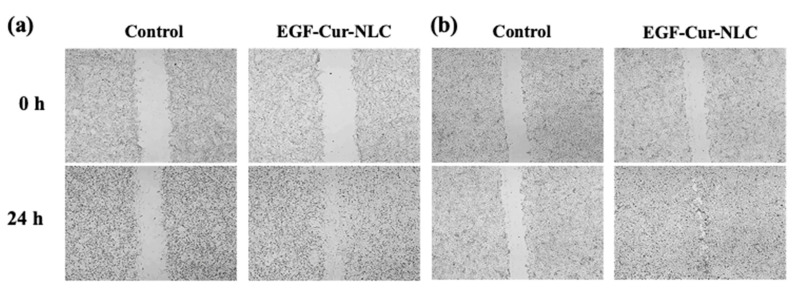
Cell migration assay for (**a**) NIH 3T3 fibroblast and (**b**) HaCaT keratinocytes.

A migration assay was performed to mimic the healing process using NIH 3T3 fibroblasts and HaCaT keratinocytes. The control group was treated with serum-free DMEM. NIH 3T3 fibroblasts and HaCaT keratinocyte closed the gap faster in the presence of EGF–Cur-NLC than with the control treatment (Figure 5). The proliferation and migration assays were conducted using NIH 3T3 fibroblasts and HaCaT keratinocytes. Fibroblasts and keratinocytes are the major components of granulation tissue and epithelial barrier. They are the main cells involved wound closure [36]. In the proliferation assay, EGF in the NLC preparation did not show loss of bioactivity; EGF–Cur-NLC efficiently induced cell proliferation. Fibroblasts and keratinocytes migration assays are suitable for mimicking in vivo cell migration during wound healing [37]. Fibroblasts migration at the wound site lead to the production of matrix components [38]. The migration of keratinocytes is associated with the process of re-epithelialization [39]. The migration assay indicated that EGF–Cur-NLC promoted cell migration and, thus, gap closure, faster than the control treatment. In summary, cell proliferation was slightly higher in the presence of EGF–Cur-NLC than in the presence of EGF–Cur-Mix, although there was no significant difference between the groups. This result might be due to the sustained release of EGF from EGF–Cur-NLC. In the in vitro release test, EGF and Cur showed sustained release profiles. Therefore, this result demonstrates that EGF–Cur-NLC affects re-epithelialization by stimulating fibroblasts and keratinocytes in chronic wounds. Moreover, during the experiments, there was no cytotoxic effect at the treatment concentration.

### 2.5. Antioxidant Activity of EGF–Cur-NLC

**Figure 6:**
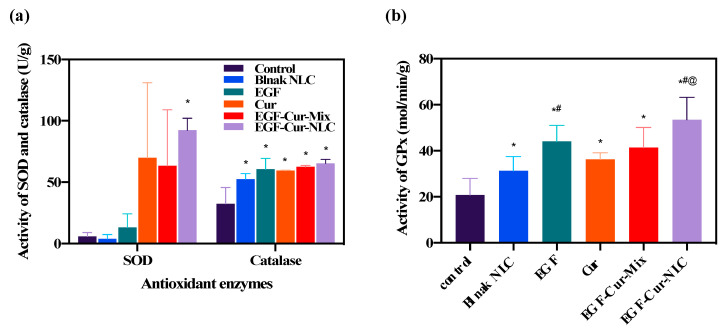
Antioxidant enzymes activity after treatment with the different formulations. (**a**) Superoxide dismutase (SOD), catalase, and (**b**) glutathione peroxidase (GPx) activities in wounded tissues; * *p* < 0.05 compared to the control, # *p* < 0.05 compared to blank-NLC, @ *p* < 0.05 compared to EGF–Cur-Mix.

To evaluate the antioxidant effect of EGF–Cur-NLC, superoxide dismutase (SOD), catalase, and glutathione peroxidase (GPx) levels were evaluated after the application of the formulations to wounded tissue. Figure 6 illustrates the activities of antioxidant enzymes. Treatments with Cur, EGF–Cur-Mix, and EGF–Cur-NLC increased SOD activity. However, only the EGF–Cur-NLC treatment showed a statistically significant increase if compared to the control group (Figure 6a). When catalase activity was evaluated in wounded tissue, blank-NLC, EGF, Cur, EGF–Cur-Mix, and EGF–Cur-NLC significantly increased the catalase level if compared to the control group. A similar pattern was found for GPx (Figure 6b). However, EGF and EGF–Cur-NLC induced a higher GPx activity than blank-NLC. GPx activity in the presence of EGF–Cur-NLC was significantly higher than in the presence of EGF–Cur-Mix. In summary, SOD, catalase, and GPx activities were increased after the application of EGF–Cur-NLC. Also, considering the GPx activity, EGF–Cur-NLC potentiated the antioxidant enzyme activity if compared to EGF–Cur-Mix. this indicates that NLC improved the activity of antioxidant enzymes, in particular SOD and GPx, in wounded tissue.

### 2.6. In Vivo Wound Healing Study

**Figure 7:**
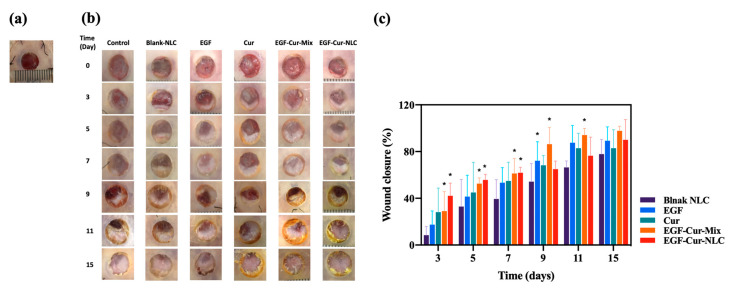
Chronic-wound healing effect of blank-NLC, EGF, Cur, EGF–Cur-Mix, and EGF–Cur-NLC in diabetic rats. (**a**) Wound image with silicone splint, (**b**) wound images after the application of the formulations at day 0 and 3, (**c**) %wound closure calculated as percentage area of the initial wound; * *p* < 0.0.5 compared to the control group.

The diabetic rat model induced by an injection of streptozotocin was used. Our team used the diabetic rat model in a previous study. According to this previous study, a glucose level >200 mg/dL was maintained over three weeks after the injection of streptozotocin [40]. The effect of EGF–Cur-NLC on chronic-wound healing was investigated in the full-thickness wound model. A biopsy punch (8 mm) was used to create a uniform wound size. Silicone splints were sutured and fixed to prevent wound contraction. On day 3, the EGF–Cur-NLC group (42.11 ± 10.83%) showed significant closure compared to all other groups (10.39 ± 6.91 for the untreated control group, 8.41 ± 7.48 for the blank-NLC group, 17.44 ± 11.71 for the free-EGF group, 21.37 ± 21.47 for the free-curcumin group, and 29.00 ± 16.76 for the EGF–Cur-Mix group) (Figure 7). On days 9 and 11, the EGF–Cur-Mix group (86.31 ± 14.21 by day 9 and 94.14 ± 5.76 by day 11) showed a higher wound closure in comparison to the EGF–Cur-NLC group (64.95 ± 6.87 by day 9 and 76.46 ± 15.93 by day 11. However, all groups did not statistically differ with respect to wound contraction after 15 days. The wound healing process is a natural healing process, and wounds are healed naturally even without drug treatment. The natural healing time varies depending on the size or location of the wound and the physiological conditions. Therefore, a method to evaluate the wound healing time or the half-healing time has been reported [41,42]. We evaluated the half-healing time after treatment with the different formulations. When the data of wound closure (%) was fitted by the sigmoidal Emax model, the time (day) to achieve 50% wound closure was 8.02, 6.43, 5.84, 5.24, and 4.26 days for blank-NLC, EGF, Cur, EGF–Cur-Mix, and EGF–Cur-NLC, respectively (Figure 7). The healing time (9 and 11 days in EGF-Cur-Mix and EGF-Cur-NLC, respectively) and half-healing time for EGF–Cur-NLC was shorter than that for EGF–Cur-Mix. From the in vivo wound healing assay, it was concluded that the EGF–Cur-NLC accelerated wound healing in the early stages of the healing process. This could be attributed to the following three reasons: (i) Many researchers have attempted to use EGF for wound healing, but most have failed because of high proteases activities [43]. Similarly, wound closure in the presence of free EGF was not significantly different from that in the untreated control group in this study. Therefore, the encapsulation process enhanced the stability of EGF by protecting it from wound proteases and oxidative stress; (ii) A controlled release of EGF can provide a sustained EGF concentration to the receptor, which can continuously stimulate cell proliferation and migration [44]; (iii) THE EGF–Cur-NLC group presented higher antioxidant levels than the other groups. Higher antioxidant levels might be related to increased curcumin solubility in the NLC formulation [45]. NLC improved the antioxidant activity of EGF. Therefore, accelerated wound closure by EGF–Cur-NLC was due to the antioxidant properties of Cur and EGF. Also, accelerated wound closure can prevent infection, drying, and tissue trauma at the wound site [46].

However, the wound closure rate with EGF–Cur-Mix increased more than that with EGF–Cur-NLC over time. This may be due to the following reasons: the samples were treated with a time interval of 2 days. An in vitro release study showed that about 80% and 40% of EGF and curcumin were released from EGF–Cur-NLC in 48 h. In the case of EGF–Cur-Mix, a mixture of free EGF and curcumin was released at once at the wound site. The wound in the untreated group was also repaired. However, EGF–Cur-NLC accelerated wound closure and improved the antioxidant activity in the wound.

Li et al. (2016) developed an EGF- and curcumin-co-encapsulated nanoparticle/hydrogel system and evaluated its wound healing effect [47]. We developed EGF–Cur-NLC and focused on the antioxidant effect and wound healing effect of this formulation. The antioxidant effect helps to control oxidative stress in the wound and accelerates wound healing. Therefore, the antioxidant effect in a wound should be evaluated to clarify the mechanism by which a formulation promotes wound healing. Our work showed that EGF–Cur-NLC accelerated wound healing by inducing an antioxidant effect and by stimulating the migration/proliferation of keratinocytes and fibroblasts. However, to clarify the mechanism of action of EGF–Cur-NLC, the coverage of the granulation tissue and epithelial tissue should be evaluated in vivo. Moreover, the evaluation of collagen disposition will also clarify this issue.

In summary, a combination therapy with EGF and curcumin accelerated wound closure. NLC for co-delivery of EGF and curcumin was effective for the treatment of chronic wounds. Thus, EGF–Cur-NLC is a promising therapeutic agent for chronic-wound healing.

## 3. Materials and Methods

### 3.1. Materials

EGF, curcumin, 3-(4,5-dimethylthoazol-2yl)-2,5-diphenyl-2*H*-tetrazolium bromide (MTT), and ethylenediaminetetraacetic acid (EDTA) were obtained from Sigma-Aldrich (St. louis, MO, USA). Dimethyl sulfoxide (DMSO), olive oil, oleic acid, glyceryl monostearate (GMS), Tween 80, and Tween 20 were purchased from Samchun Chemical (Pyungtaek, Korea). Stearic acid was provided from Daejung Chemical (Cheongwon, Korea). Precirol ATO 5, Compritol 888 ATO, Capryol 90, Capryol PGMC, Labrafac CC, Labrafil M 1944 CS, Peceol, Lauroglycol FCC, Labrafac WL 1349, and Cremophor EL were obtained from Gattefossé (Saint Priest, Cedex, France). Lutrol F-68 (poloxamer 188) and Lutrol F-127 (poloxamer 407) were provided by BASF (Ludwigshafen, Germany). NIH 3T3 and HaCaT cells were obtained from the Korean Cell Line Bank (Seoul, Korea). Dulbecco’s modified Eagle’s medium (DMEM), fetal bovine serum (FBS), penicillin–streptomycin, and trypsin–EDTA were purchased from Gibco BRL (Gaithersburg, MD, USA). Methanol was obtained from JT baker (Phillipsburg, NJ, USA).

### 3.2. Screening of Liquid Lipids and Surfactants

The measurement of curcumin solubility in liquid lipids and surfactants was performed to select the optimal formulation. Briefly, an excess amount of curcumin was mixed with 0.5 mL of various liquid lipids. To achieve the equilibrium, the various liquid lipids with curcumin were mixed using an end-over-end shaker (EYELA, Tokyo, Japan) at 10 rpm. After 72 h of equilibrium, the samples were centrifuged at 15,000× *g* for 10 min, and undissolved curcumin was removed. The solubility of curcumin was calculated by HPLC with a UV detector (LC-2030C HPLC system, Shimadzu, Kyoto, Japan). Curcumin solubility in various 1% (*w*/*v*) surfactants was evaluated in the same way.

### 3.3. Preparation of EGF–Cur-NLC

**Figure 8:**
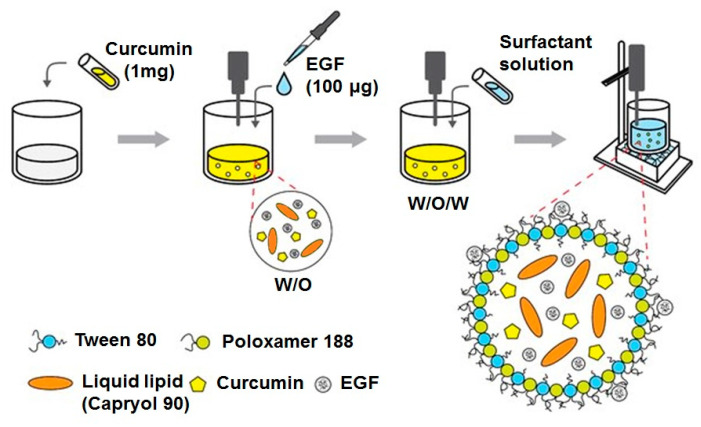
Preparation process of EGF–Cur-NLC.

EGF–Cur-NLC was prepared by the modified w/o/w double-emulsion method (Figure 8). Brifely, the weighted solid lipid (Precirol ATO 5) and liquid lipid (Capryol 90) were mixed with 200 µL of a dichloromethane solution containing curcumin (1 mg). The mixture was emulsified with an EGF aqueous solution (100 µg) using ultrasonication (Vibra-Cell, Sonics & Material Inc., Newtown, CT, USA; amplitude 20%, 15 s). This step produced a w/o emulsion; then, the w/o emulsion was mixed to a water phase containing a 1% surfactant solution (*w*/*v*, 0.5% poloxamer 188 and 0.5% Tween 80 solution) and sonicated for 30 s. The final w/o/w emulsion was homogenized (T 25 digital ULTRA-TURRAX^®^, IKA, Wilmington, NC, USA) at 16,000 rpm for 5 min in an ice bath. For the preparation of blank-NLC, the same method was used in in the absence of EGF and curcumin. EGF–Cur-NLC and blank-NLC were freeze–dried using a lyophilizer (FD-1000, EYELA, Tokyo, Japan).

### 3.4. Physicochemical Properties of EGF–Cur-NLC

To assess the particle size and PDI of EGF–Cur-NLC, samples diluted with distilled water were evaluated using dynamic light scattering (DLS, ELS-8000, Otsuka Electronics, Osaka, Japan). Measurements were performed for 50 times, and the average value was obtained.

Differential scanning calorimetry (DSC) analysis was performed using a DSC N-650 thermal analyzer (Scinco, Seoul, Korea). In brief, samples were weighed (2 mg) in aluminum pans, and the analysis was carried out by heating the samples from 30 °C to 250 °C at a heating rate of 10 °C min^−1^ under a nitrogen flow.

Samples for X-ray diffraction (XRD) analysis were examined using a D/Max-2200 Ultima/PC (Rigaku, Tokyo, Japan) with Ni-filtered Cu-Kα radiation (40 kV, 40 mA). The samples were investigated over a 2θ range from 5° to 60° with step size of 0.02°/s.

### 3.5. Determination of Drug Content and EE

The ultrafiltration method was used to evaluate the EE of EGF–Cur-NLC. In brief, 0.5 mL of EGF–Cur-NLC was added in the ultrafilter device (100 kDa, Amicon Ultra, Millipore, Billerica, MA, USA) and centrifuged at 14,000× *g* for 30 min. The amounts of curcumin and EGF in the soup were evaluated to calculate the EE.

The amount of un-encapsulated curcumin was analyzed by an HPLC system with a C18 column (Phenomenex Gemini, 5 μm, 110 Å, 250 × 4.6 mm). The mobile phase consisted of acetonitrile (ACN) and 0.1% formic acid (50/50, *v*/*v*), and the flow rate was set at 1.3 mL/min. For the analysis, 20 µL of sample was injected, and UV detection was conducted at 425 nm. The amount of un-encapsulated EGF was analyzed using an enzyme-linked immunosorbent assay (ELISA) kit (R&D Systems, Minneapolis, MN, USA). EE was calculated by the following equation:

EE (%) = (Total amount of drug − Amount of free drug)/(Total amount of drug) × 100(1)

### 3.6. In Vitro Release Study of EGF–Cur-NLC

The in vitro release study was performed using Franz diffusion cells, comparing the release rate of EGF from EGF–Cur-NLC to that from a solution of free EGF and curcumin. Briefly, a 100 kDa dialysis membrane was clamped between a donor and a receptor chamber. The EGF–Cur-NLC formulation or solution of free EGF and curcumin (0.5 mL) was placed in the donor chamber. The receptor chamber was filled with receptor fluid made of phosphate buffer saline (PBS, pH 7.4) or 50% ethanol solution. Ethanol was used to lead the complete release of curcumin. The receptor chamber was maintained at about 37 ± 0.5 °C. At predetermined time intervals (1, 4, 6, 17, 26, and 48 h), samples of the receptor chamber were collected and replaced with equal volumes of fresh medium to maintain sink conditions. The concentrations of EGF and curcumin were analyzed by ELISA and HPLC.

To determine the mechanism of drug release, the Korsmeyer–Peppas model was used. Drug release was determined with the following equation:

M_t_/M_∞_ = *k*t (2)

where *k* is a constant. Based on the diffusional index (*n*) values, the mechanism of release was determined (*n* < 0.45, quasi-Fickian diffusion; *n* = 0.45, Fickian release; 0.45 < *n* < 0.89, non-Fickian release (anomalous release); *n* = 0.89, case II release (zero-order release); *n* > 0.89, super case II release).

### 3.7. In Vitro Cell Study of EGF–Cur-NLC

#### 3.7.1. Cell Culture Condition

To evaluate cell proliferation and migration, fibroblasts (NIH 3T3 cell) and keratinocytes (HaCaT) were used. Cells were sub-cultured in DMEM supplemented with 10% FBS, penicillin (100 units/mL), and streptomycin (100 μg/mL) at 37 °C with a 5% CO_2_ atmosphere.

#### 3.7.2. Cell Proliferation Assay

The MTT assay was used to evaluate cell proliferation of NIH 3T3 and HaCaT cells. To perform the study, the cells were seeded at a density of 1 × 10^4^ per well in a 24-well plate and incubated for 24 h. Then, the following treatments were performed: (1) control (serum-free DMEM), (2) blank-NLC, (3) EGF–Cur-Mix (mixture of 10 ng/mL free EGF and 100 ng/mL free curcumin), (4) EGF–Cur-NLC (10 ng/mL EGF and 100 ng/mL curcumin). After incubation for 12 h, 24 h, and 48 h, 30 μL of MTT solution was added to the wells. After 3 h of incubation, the mixture was removed, and 200 μL of DMSO was added. Finally, the absorbance was measured at 560 nm by using a micro-plate reader (Sunrise, Tecan, Austria). Herein, we used EGF at doses of 10 ng/mL. This concentration was selected based on the literature [48]. According to the literature, EGF in the concentration range of 2–20 ng/mL induced concentration-dependent keratinocyte migration and proliferation.

#### 3.7.3. Cell Migration Assay

NIH 3T3 fibroblasts and HaCaT keratinoctyes were seeded at 1 × 10^6^ cells per well in 12-well plates and cultured overnight to form a confluent monolayer. A scratch was created with a sterile pipette tip, and all wells were washed with cold PBS. PBS was removed and replaced with (1) control (serum-free DMEM), (2) blank-NLC, (3) EGF–Cur-Mix (mixture of 10 ng/mL free EGF and 100 ng/mL free curcumin), (4) EGF–Cur-NLC (10 ng/mL EGF and 100 ng/mL curcumin). Cell images were obtained using a microscope (Nikon Eclipse Ti; Nikon Instruments Inc., Melville, NY, USA).

### 3.8. In Vivo Study

#### 3.8.1. Animals

Male Sprague-Dawley rats aged 7 weeks were used obtained from Samtako (Osan, Korea). Rats were acclimated in cages for 1 week and placed on a 12:12 h light/dark cycle. Feed and water were provided ad libitum. Before the induction of chronic wounds, rats received streptozotocin via the tail vein at a dose of 45 mg/kg to induce diabetes. After 1 week from the injection, blood glucose levels were measured using a blood glucose meter (Accu-Chek^®^ 114 Active, Hoffmann-La Roche, Basel, Switzerland). Only rats with a blood glucose level above 300 mg/dL were selected and used in further experiments. All experiments were carried out in accordance with guidelines established by the Chungnam National University Institutional Animal Care and Use Committee and approved by the Local Ethical Committee of Chungnam National University (No. CNU-01149).

#### 3.8.2. Diabetic Wound Healing Animal Model

Diabetic rats were used to assess the wound healing effect of EGF–Cur-NLC. In brief, a day before the experiment, hair was shaved from a dorsal area, and the shaved part was wrapped with a dressing for the protection of skin. On the day of the experiment, two portions of the back skin including deep layers (epidermis, dermis, and superficial fat) were removed using a skin biopsy punch (8 mm diameter) under ether anesthesia. To fix the shape of the wound, a donut-shaped silicone splint was sutured on the skin (Figure 7a). The wounds were treated with the 5 formulations: (a) blank-NLC, (b) 5 μg of free EGF, (c) 50 μg of free curcumin, (d) EGF–Cur-Mix (mixture of 5 μg free EGF and 50 μg free curcumin), (e) EGF–Cur-NLC (5 μg EGF and 50 μg curcumin). Rats in the received two wounds: a wound on the right side that was treated with one of the formulations and an untreated wound on the left as a control. Formulations were administered on the wound area with 80 μL vehicle (3% carboxymethylcellulose, *w*/*v*) using a micropipette. The treated skin was wrapped with film dressing and an elastic bandage (Tegaderm™ and Coban™, 3M, Sao Paulo, MN, USA).

Each wound area was photographed on days 0, 3, 5, 7, 9, 11, 15. The degree of wound closure was calculated using ImageJ^®^ software (Aspire Software International, Leesburg, VA, USA). The wound closure was calculated as follows:

Wound closure (%) = (A_0_ − A_t_)/A_0_ (3)

where A_0_ and A_t_ are the wound area on days 0 and t, respectively. When the wound area was equal to that on day 0, wound repair was considered complete.

On day 16, all animals were killed, and the wounded tissue was collected for the evaluation of antioxidant enzymes’ activities, including SOD, catalase, and GPx. In brief, tissue was homogenized in cold PBS (pH 7.4) at 4 °C. The samples were centrifuged at 12,000 rpm for 10 min at 4 °C. Then, the supernatants were stored at −80 °C, and the activity of antioxidant enzymes was measured using ELISA kits (SOD, Thermo Fisher Scientific Inc., Rockford, IL, USA; catalase, Thermo Fisher Scientific Inc., Rockford, IL, USA; GPx, Cayman Chemical, Ann Arbor, MI, USA).

#### 3.8.3. Statistical Analysis

Data were expressed as mean ± standard deviation (SD). Differences between groups of samples were analyzed by Student’s t-test and one-way ANOVA; *p* < 0.05 was considered statistically significant.

## 4. Conclusions

In this study, EGF–Cur-NLC was successfully developed by the w/o/w double-emulsion method. EGF–Cur-NLC spherical particles showed a small particle size (331.8 nm) and high encapsulation efficiency (81.1 and 99.4% for EGF and Cur, respectively). In cell studies, EGF–Cur-NLC improved the migration and proliferation of fibroblasts and keratinocytes. Also, EGF–Cur-NLC increased the activity of antioxidant enzymes, in particular, SOD and GPx. When the half-healing time of EGF–Cur-NLC was evaluated, EGF–Cur-NLC showed a shorter half-healing time if compared to EGF, Cur, and EGF–Cur-Mix. Therefore, EGF–Cur-NLC can accelerate wound closure, thus decreasing the risk of bacterial infection. We conclude that EGF–Cur-NLC could be a promising therapeutic agent for chronic-wound healing.

## Figures and Tables

**Table 1:** (**a**) Composition of various formulations, (**b**) physicochemical properties of formulations according to the ratio of solid and liquid lipids. EGF, epidermal growth factor, PDI, polydispersity index, EE, encapsulation efficiency.

(**a**)				
Formulation	Precirol ATO 5 (mg)	Capryol 90 (mg)	EGF (μg)	Curcumin (mg)
F1	70	30	100	1
F2	60	40	100	1
F3	50	50	100	1
(**b**)				
Formulation	Particle Size (nm)	PDI	EE of EGF (%)	EE of Curcumin (%)
F1	331.8 ± 29.1	0.31 ± 0.03	81.1 ± 0.8	99.4 ± 0.1
F2	394.3 ± 17.5	0.36 ± 0.01	79.0 ±1.9	99.2 ± 0.4
F3	509.2 ± 18.3	0.34 ± 0.02	79.8 ± 1.3	99.2 ± 0.0

**Table 2:** Parameters of Korsmeyer–Peppas model for drug release from EGF–Cur-NLC.

Drug	*n*	k	R^2^	Release Mechanism
EGF	0.4	0.28	0.9	Quasi-Fickian diffusion
Cur	0.37	0.4	0.89	Quasi-Fickian diffusion
EGF in EGF–Cur-NLC	0.26	0.37	0.91	Quasi-Fickian diffusion
Cur in EGF–Cur-NLC	0.31	0.31	0.85	Quasi-Fickian diffusion
